# Association between rs2303861 polymorphism in *CD82* gene and non-alcoholic fatty liver disease: a preliminary case-control study

**DOI:** 10.3325/cmj.2019.60.361

**Published:** 2019-08

**Authors:** Parham Habibzadeh, Behnam Honarvar, Mohammad Silawi, Shima Bahramjahan, Azar Kazemi, Mohammad Ali Faghihi, Kamran Lankarani

**Affiliations:** 1 Student Research Committee, Shiraz University of Medical Sciences, Shiraz, Iran; 2 Health Policy Research Center, Institute of Health, Shiraz University of Medical Sciences, Shiraz, Iran; 3 Persian BayanGene Research and Training Center, Shiraz, Iran; 4 Transplant Research Center, Shiraz University of Medical Sciences, Shiraz, Iran; 5 Center for Therapeutic Innovation, Department of Psychiatry and Behavioral Sciences, University of Miami Miller School of Medicine, Miami, FL, USA

## Abstract

**Aim:**

To investigate the genetic factors involved in the development of non-alcoholic fatty liver disease (NAFLD) and its sequelae in a Middle Eastern population.

**Methods:**

This genetic case-control association study, conducted in 2018, enrolled 30 patients with NAFLD and 30 control individuals matched for age, sex, and body mass index. After quality control measures, entire exonic regions of 3654 genes associated with human diseases were sequenced. Allelic association test and enrichment analysis of the significant genetic variants were performed.

**Results:**

The association analysis was conducted on 27 NAFLD patients and 28 controls. When Bonferroni correction was applied, NAFLD was significantly associated with rs2303861, a variant located in the *CD82* gene (*P* = 2.49 × 10^−7^, adjusted *P* = 0.0059). When we used Benjamini-Hochberg adjustment for correction, NAFLD was significantly associated with six more variants. Enrichment analysis of the genes corresponding to all the seven variants showed significant enrichment for miR-193b-5p (*P* = 0.00004, adjusted *P* = 0.00922).

**Conclusion:**

A variant on *CD82* gene and a miR-193b expression dysregulation may have a role in the development and progression of NAFLD and its sequelae.

Non-alcoholic fatty liver disease (NAFLD) is the most common liver disease in many parts of the world and an important global health concern (1-4). As a hepatic manifestation of metabolic syndrome, it is closely associated with obesity, insulin resistance, and dyslipidemia (5). NAFLD prevalence is steadily on the rise due to a global increasing trend in obesity incidence (5,6) and within the next decade NAFLD is predicted to replace hepatitis C as the leading indication for liver transplantation in the United States (1).

The main causes of mortality among patients with NAFLD are malignancies and cardiovascular diseases (7,8). The condition is an independent risk factor for hepatocellular carcinoma (HCC), which was previously thought to require liver cirrhosis as its precursor but has been recently described in patients with simple hepatic steatosis and no sign of inflammation or fibrosis (9,10). NAFLD is also closely associated with an increased risk of colorectal cancer (11,12), as well as with myocardial remodeling, thus playing an important role in the development of heart failure (13,14). The observed association between NAFLD, HCC, colorectal cancer, and heart failure implies that there is a possible underpinning defect in cellular processes that link cell metabolism to cell division and metabolite trafficking.

The underlying risk factors for NAFLD (eg, obesity and diabetes mellitus) are associated with numerous genetic polymorphisms (15-17). Genetic factors could also in part explain the extreme variation in the worldwide NAFLD prevalence (6) and considerable inter-individual variability in disease severity, morbidity, and mortality (18). NAFLD was found to be significantly associated with SNP rs738409 (I148M) in *PNPLA3* gene on chromosome 22, encoding an enzyme responsible for the hydrolysis of triacylglycerols in adipocytes; individuals homozygous for this allele had more than twice as much hepatic fat content as non-carriers (19). Variants in other genes (eg, *MBOAT7*, *TM6SF2*, etc) (20), as well as dysregulated expression of several micro-RNAs (miRNAs), were also found to contribute to NAFLD pathogenesis (21). For instance, miR-33a/b modulates the risk of metabolic syndrome by regulating various metabolic pathways (22).

To the best of our knowledge, no genetic case-control association study has so far been conducted on NAFLD in the Middle Eastern population, despite the fact that the underlying genetic factors of NAFLD and their relative contributions might differ from those in other populations. Therefore, after controlling for traditional risk factors, we investigated the underlying genetic factors involved in the development of NAFLD and its sequelae in an Iranian population.

## Materials and methods

### Study population

The study included 30 patients with NAFLD (17 female) and 30 healthy controls (17 female) matched for age, sex, and body mass index (BMI), who were randomly selected (with a random number generator) from participants of a cross-sectional population-based study previously conducted in Shiraz (23). Briefly, the previous study had enrolled 542 adult unrelated participants randomly selected from the general population through a proportional cluster random sampling. They had been interviewed to obtain demographic information and physically examined by a medical doctor. Participants with alcohol consumption, participants serologically positive for hepatitis B or hepatitis C, and those identified through the interview to have hepatic steatosis due to other competing etiologies (eg, drugs, bariatric surgery, etc) had been excluded. NAFLD had been ultrasonographically diagnosed by an experienced radiologist unaware of the patients’ clinical information according to a validated protocol (24).

The current study included only patients with confirmed moderate-to-severe hepatic steatosis. The absence of hepatic steatosis in the control group was also documented by ultrasonography.

This study was conducted in compliance with the Declaration of Helsinki. Written informed consent was obtained from all study participants who had been assured that their data would be kept confidential. The study was approved by the Research Ethics Committee of Health Policy Research Center affiliated to Shiraz University of Medical Sciences Ethics Committee.

### Clinical and laboratory evaluation

Weight, height, waist circumference, hip circumference, and blood pressure had been measured and BMI was calculated (23). Fasting venous blood samples were obtained, serum was separated, and fasting glucose, aspartate aminotransferase (AST), alanine aminotransferase (ALT), triacylglycerols, high-density lipoprotein cholesterol, low-density lipoprotein cholesterol, hepatitis B surface antigen, and hepatitis C virus antibodies were measured with an automated analyzer (CS-1200 Auto-Chemistry Analyzer, DIRUI Industrial Co, Changchun, China).

### DNA preparation, genotyping, and quality control

Genomic DNA was extracted from EDTA-anticoagulated peripheral whole blood collected from each individual with QIAamp DNA isolation mini kit (QIAGEN, Hilden, Germany) by salting out technique. The quality of isolated DNA specimens was assessed by QUBIT 3.0 fluorometer (Thermo Fisher Scientific, Waltham, MA, USA). The entire exonic regions of 3654 genes associated with human diseases (inherited disease panel, Agilent Technologies, Santa Clara, CS, USA) were sequenced above 70 × coverage on an Illumina NextSeq 500 platform (Illumina, San Diego, CA, USA). A total of 54 100 variants located in genomic loci of these genes were identified. Target Enrichment for Illumina Multiplexed sequencing protocol (v. D0, November 2015) with SureSelect^QXT^ (Agilent Technologies) was used for individual library preparation and sample pooling. The following quality control measures were performed in PLINK, v. 1.07 (Free Software Foundation Inc., Boston, MA, USA) (25): single nucleotide polymorphisms (SNPs) with minor allele frequency of <0.05, those with significant deviation from the Hardy-Weinberg equilibrium (*P* < 10^−5^), and those genotyped for less than half of the studied population were excluded from analysis. All samples were screened for known damaging variants in the *ATP7B*, *HFE*, and SE*RPINA1* genes corresponding to Wilson’s disease, hemochromatosis, and α_1_-antitrypsin deficiency, respectively. Samples with damaging mutations were excluded from the study.

### Statistical analysis

The normality of distribution of continuous variables was tested with the one-sample Kolmogorov-Smirnov test. The variables are presented as mean ± standard deviation. Significance of differences between two groups was tested with the *t* test for independent samples. The analyses were conducted in IBM SPSS, version 20 (IBM, Armonk, NY, USA).

The association between SNP genotype and NAFLD was assessed with the allelic association test in PLINK, which is a simple χ^2^ test for association based on a 2 × 3 case-control genotype counts contingency table (25,26). The obtained *P* values were adjusted with Bonferroni correction for multiple comparisons, with the level of significance set at *P* < 2.1 × 10^−6^. Manhattan plot was drawn in Haploview (27). Logistic regression analysis was performed in PLINK using an additive genetic model adjusting for age, sex, and BMI. Genotypes for SNPs with significant *P* values were recoded as 0, 1, or 2, according to the number of alternative alleles present. Multiple linear regression analysis, performed in SPSS, assessed the effect of the SNP’s alternative alleles that were found significant in allelic association test on clinical and biochemical parameters after adjustment for age, sex, and BMI. False discovery rate for each association was calculated with the Benjamini-Hochberg method (28). SNPs with a false discovery rate <0.05 were included in the enrichment analysis. Information about overlapping or nearest gene corresponding to the associated SNPs was obtained by an SNP annotation tool (29). Enrichment analysis was carried out with Enrichr on miRTarBase database (30,31). Significantly enriched miRNAs were identified from the list of genes corresponding to the SNPs that were found to be significantly different between patients and controls with the Fisher exact test in Enrichr.

## Results

After quality control, 55 individuals – 27 patients with NAFLD and 28 controls – and 23 732 SNPs were included in the association analysis. Patients with NAFLD had significantly higher mean fasting blood glucose and serum ALT (*P* < 0.05) (Table 1). The groups did not differ in any other parameter.

**Table 1:** Clinical and biochemical characteristics of cases and controls

Parameter, mean and standard deviation	NAFLD group (n = 27)	Control group (n = 28)	*P*
Age (years)	52.2 (10.1)	51.0 (9.0)	0.638
Body mass index (kg/m^2^)	28.0 (5.3)	27.2 (5.1)	0.569
Fasting blood glucose (mg/dL)	109.6 (40.4)	90.4 (14.7)	0.026
Triacylglycerols (mg/dL)	159.8 (71.0)	158.29 (75.6)	0.940
Low-density lipoprotein cholesterol (mg/dL)	106.8 (35.6)	117.7 (27.3)	0.208
High-density lipoprotein cholesterol (mg/dL)	45.2 (8.5)	49.7 (11.0)	0.093
Aspartate aminotransferase (IU/L)	28.0 (12.1)	24.1 (13.6)	0.273
Alanine aminotransferase (IU/L)	34.0 (18.5)	20.2 (8.7)	0.001
Systolic blood pressure (mm Hg)	120.6 (16.8)	120.1 (22.1)	0.933
Diastolic blood pressure (mm Hg)	77.9 (8.1)	78.5 (8.2)	0.807
Waist circumference (cm)	96.2 (18.6)	95.0 (11.5)	0.763
Hip circumference (cm)	103.7 (18.9)	104.1 (8.5)	0.911

**Figure 1:**
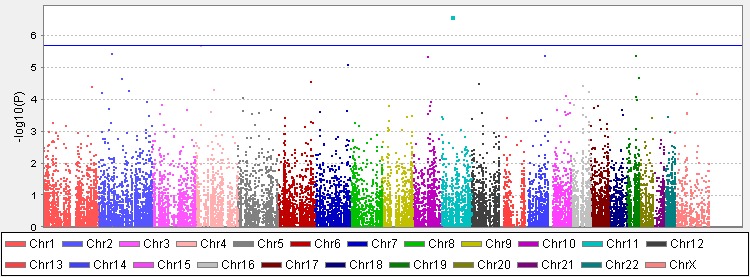
The Manhattan plot based on the data obtained from the allelic association test. The x-axis represents the chromosomal location of the studied single nucleotide polymorphisms (SNPs). The y-axis is –*log*_10_(*P* value). The solid horizontal line corresponds to the Bonferroni-adjusted significance threshold, a *P* value of 2.1 × 10^−6^.

When Bonferroni correction was applied, NAFLD was significantly associated with rs2303861, located on *CD82* gene (Figure 1, Table 2). After adjusting for age, sex, and BMI, the alternative allele of ‘G’ was associated with a 9-fold increase in the risk of developing NAFLD compared with the wild type ‘A’ (odds ratio 9.07, 95% confidence interval 1.92-42.81). When Benjamini-Hochberg method was applied, six more SNPs were found to have false discovery rates of <0.05 (Table 2), meaning that they were also associated with NAFLD development.

**Table 2:** Leading single nucleotide polymorphisms (SNPs) associated with non-alcoholic fatty liver disease (NAFLD) identified by the allelic association test. The adjusted odds ratio (OR) and confidence interval (CI) were obtained in logistic regression analysis with an additive genetic model adjusting for age, sex, and body mass index

Chromosome	SNP ID	Position	Gene	Wild/alternative allele	*P* (unadjusted)	*P* (Bonferroni correction)	FDR-BH*	Adjusted OR (95% CI)
11	rs2303861	44618466	CD82	A/G	2.49 × 10^−7^	0.0059	0.0059	9.07 (1.92-42.81)
4	rs13116873	26362678	RBPJ	C/G	2.12 × 10^−6^	0.0502	0.0181	13.91 (2.38-81.45)
2	rs4671501	63404754	WDPCP	A/C	3.69 × 10^−6^	0.0875	0.0181	0.10 (0.02-0.58)
14	rs1742546	91417155	CCDC88C	G/A	4.15 × 10^−6^	0.0984	0.0181	0.25 (0.10-0.61)
19	rs2075754	41871176	RPS19	T/C	4.39 × 10^−6^	0.1043	0.0181	4.96 (1.57-15.66)
10	rs3752752	71695444	CDH23	T/C	4.57 × 10^−6^	0.1085	0.0181	4.52 (1.71-11.92)
7	rs740949	148808972	EZH2	A/G	8.37 × 10^−6^	0.1987	0.0284	0.19 (0.06-0.57)

*False discovery rate obtained by Benjamini-Hochberg method.

Multiple linear regression analysis did not show any significant association between the seven SNP alleles and clinical and biochemical parameters, taking into account the multiple statistical tests performed (Table 3). Enrichment analysis of the genes corresponding to the seven SNPs found in the association study showed significant enrichment for miR-193b-5p (*P* = 0.00004, adjusted *P* = 0.00922).

**Table 3:** Association between significant single nucleotide polymorphisms (SNPs) and clinical and biochemical variables in non-alcoholic fatty liver disease (NAFLD) patients and controls. Coefficients (B) and their standard errors (SE) and *P* values were obtained from multiple linear regression analysis adjusted for age, sex, and body mass index

SNP	Subject	Aspartate aminotransferase		Alanine aminotransferase		Fasting blood glucose		Triacylglycerols		Low-density lipoprotein		High-density lipoprotein		Systolic blood pressure		Diastolic blood pressure		Waist circumference		Hip circumference	
B (SE)	*P*	B (SE)	*P*	B (SE)	*P*	B (SE)	*P*	B (SE)	*P*	B (SE)	*P*	B (SE)	*P*	B (SE)	*P*	B (SE)	*P*	B (SE)	*P*
rs2303861	Control	0.09 (3.89)	0.982	-1.71 (2.51)	0.502	-1.89 (3.82)	0.625	-2.22 (22.78)	0.923	-2.99 (8.18)	0.718	2.28 (3.00)	0.454	12.80(4.88)	0.015	1.90(2.22)	0.401	3.15 (2.31)	0.185	-0.48(1.55)	0.761
	NAFLD	-2.63 (2.82)	0.362	-2.76 (3.69)	0.463	-2.15 (8.52)	0.803	-7.94 (16.76)	0.64	-6.14 (7.07)	0.395	0.78 (1.90)	0.687	1.97 (3.17)	0.542	0.52 (1.87)	0.782	-1.12 (3.06)	0.716	-1.07 (3.05)	0.729
rs13116873	Control	7.37 (8.55)	0.397	10.78 (5.20)	0.049	-0.64 (8.58)	0.941	17.39 (50.81)	0.735	-2.86 (18.33)	0.877	-0.80 (6.78)	0.907	21.55 (11.60)	0.076	2.41 (5.03)	0.636	-6.58 (5.19)	0.218	-5.86 (3.25)	0.084
	NAFLD	0.16 (3.18)	0.96	2.08 (4.11)	0.618	17.28 (8.71)	0.06	5.06 (18.63)	0.788	3.01 (7.94)	0.709	1.39 (2.09)	0.512	4.45 (3.42)	0.206	2.35 (2.02)	0.256	4.49 (3.26)	0.182	5.17 (3.20)	0.12
rs4671501	Control	-0.09 (2.32)	0.971	-2.00 (2.93)	0.505	0.94 (3.78)	0.808	-4.01 (17.37)	0.817	-8.75 (7.15)	0.241	0.44 (3.42)	0.899	11.66 (5.99)	0.072	3.59 (2.65)	0.197	3.42 (2.05)	0.117	1.43 (1.45)	0.338
	NAFLD	-10.33 (14.69)	0.49	-26.23 (18.44)	0.171	-60.50 (41.90)	0.165	-75.54 (86.53)	0.394	-62.85 (35.00)	0.088	22.68 (7.87)	0.01	-6.05 (16.48)	0.718	-1.08 (9.66)	0.912	6.53 (8.40)	0.447	-2.91 (4.97)	0.566
rs1742546	Control	-15.89 (4.27)	0.003	-6.04 (3.56)	0.118	-3.88 (4.90)	0.446	54.14 (27.09)	0.071	-22.89 (8.47)	0.021	-1.48 (3.66)	0.695	3.05 (7.21)	0.68	-1.51 (2.66)	0.581	-5.47 (2.38)	0.042	0.90 (1.72)	0.612
	NAFLD	-1.73 (3.43)	0.622	-3.51 (4.15)	0.413	-19.42 (12.12)	0.133	-9.93 (24.47)	0.692	-7.70 (10.42)	0.473	1.47 (3.01)	0.634	-4.66 (3.59)	0.217	-2.27 (2.19)	0.319	7.50 (4.21)	0.098	-8.24 (4.23)	0.073
rs2075754	Control	-6.94 (5.16)	0.208	-6.38 (6.42)	0.344	11.28 (7.94)	0.186	80.10 (31.87)	0.031	11.31 (13.70)	0.429	-3.25 (6.59)	0.633	-15.23 (9.35)	0.135	-10.00 (4.19)	0.038	-0.173 (5.90)	0.977	-2.28 (3.82)	0.564
	NAFLD	-2.65 (2.34)	0.281	-1.41 (4.48)	0.759	-1.64 (5.29)	0.762	-10.54 (16.29)	0.531	-16.28 (6.36)	0.027	-3.20 (1.48)	0.053	4.04 (4.60)	0.399	2.80 (2.49)	0.286	2.51 (2.15)	0.267	1.42 (1.02)	0.192
rs3752752	Control	-1.93 (5.82)	0.746	0.67 (2.19)	0.766	2.56 (4.07)	0.541	-12.42 (29.84)	0.684	-8.50 (10.86)	0.448	7.85 (3.56)	0.046	-0.40 (5.88)	0.946	1.02 (1.87)	0.594	2.44 (3.18)	0.457	-0.811 (1.83)	0.665
	NAFLD	-2.69 (3.72)	0.48	-4.10 (4.13)	0.335	-1.75 (11.09)	0.876	18.09 (22.93)	0.442	11.74 (7.90)	0.157	1.24 (2.25)	0.589	-0.21 (4.33)	0.962	1.41 (2.59)	0.595	0.54 (4.33)	0.903	-1.50 (4.14)	0.722
rs740949	Control	1.13 (4.11)	0.786	0.91 (2.84)	0.753	0.82 (3.33)	0.81	0.07 (23.93)	0.998	-9.37 (7.61)	0.237	-0.35 (3.30)	0.917	0.28 (6.29)	0.965	1.97 (2.38)	0.421	0.11 (2.51)	0.965	1.73 (1.62)	0.302
	NAFLD	1.16 (4.23)	0.789	4.42 (7.78)	0.581	21.80 (16.93)	0.222	65.19 (37.44)	0.107	-22.96 (14.77)	0.146	-3.78 (3.85)	0.345	-2.35 (5.44)	0.673	-3.95 (2.88)	0.195	0.77 (2.23)	0.735	-0.42 (2.04)	0.841

## Discussion

Our study showed a significant association between NAFLD and a variant located in *CD82* gene, as well as significant enrichment for miR-193b-5p, meaning that the identified genes had significantly more targets of miR-193b-5p than expected.

One of the basic mechanisms leading to NAFLD development and progression is insulin resistance. Therefore, it is not surprising that NAFLD patients in our study had a higher fasting blood glucose level and ALT, the liver enzyme most closely associated with NAFLD (32). Since the cases and controls were matched for BMI, the differences observed between them could mainly be attributed to the processes involved in NAFLD development rather than to obesity.

*CD82*, or *KAI1*, expression is decreased or abolished in various malignant tumors (33). CD82 protein was present in about one-third of colorectal cancer tissues compared with more than half of normal mucosal tissues (34). In addition, its expression was lower in hepatocellular malignant cells compared with healthy tissue (35) and in HCC patients compared with patients with liver cirrhosis or hepatitis, or control individuals (35). Taking all this into account, our finding that a variant located in *CD82* gene was significantly associated with NAFLD might explain the increased risk of several malignancies in patients with NAFLD. However, further functional studies are required to assess the effect of this variant on *CD82* gene expression.

*CD82* null mice demonstrated enhanced bone marrow adipogenic potential, evidenced by augmented differentiation into adipocytes and enhanced expression of adipocyte differentiation markers (36). Our findings suggest that a similar mechanism may also take place in hepatocytes, leading to hepatic steatosis observed in patients with NAFLD. Furthermore, rs2303861, found in this study to be associated with NAFLD, is in linkage disequilibrium with another variant, rs7942159, located on *PNPLA2* gene, which is known to be involved in fat mobilization in adipose tissue (37,38). This might explain the effects of rs2303861 polymorphism in *CD82* observed in our study.

Expression of miR-193b is directly correlated with the secretion of adiponectin (39), a secretory protein exclusively produced by adipocytes, which increases hepatic insulin sensitivity and is inversely correlated with the presence of NAFLD and body fat content (40,41). One study also reported an inverse correlation between miR-193b-5p expression level and BMI, glucose levels, and insulin responses to a 75-g oral glucose tolerance test (42). miR-193b expression level was significantly lower in liver cancer exosome and whole tissue compared with control tissues (43). Furthermore, miR-193b down-regulation was associated with metastasis and depth of invasion in patients with liver cancer (43). Down-regulation of miR-193b was also demonstrated in colorectal cancer tissues (44) and in patients with heart failure (45).

In summary, decreased miR-193b expression and the presence of a variant in *CD82* gene might result in insulin resistance, dysregulation of adipokines, and an increase in hepatic fat content, which might all cause NAFLD and activate biological pathways leading to HCC, colorectal cancer, and heart failure. Our findings link miRNA expression to higher risk of cancer and heart disease in NAFLD patients, thus opening avenues for the treatment of NAFLD complications using miRNA blockers and miRNA mimics.

The strength of this study is the homogeneity of the studied population, use of next generation sequencing technology instead of array-based techniques, and the focus on the genetic loci associated with human diseases. The random selection of the studied sample from a population-based study can be considered an additional strength, as the studied individuals may be considered representative of the general population residing in our region.

On the other hand, the small number of participants might have decreased the statistical power of our study to detect a small effect size. Therefore, our findings require further replication studies in larger cohorts from other populations to corroborate their potential significance.

In conclusion, our study provides an insight into a possible association between decreased miR-193b expression and NAFLD development, and subsequent increased incidence of colorectal cancer, HCC, and heart failure. The existence of such association could allow the use of this miRNA in NAFLD management as a therapeutic target, disease biomarker, or method for treatment response assessment. Further studies, preferably performed in an independent cohort, should validate the associations found in this study, as well as unravel the underlying biological mechanisms involved.

Received: December 5, 2018

Accepted: June 28, 2019
